# Diagnosis and prognosis of Alzheimer's disease using brain morphometry and white matter connectomes

**DOI:** 10.1016/j.nicl.2019.101859

**Published:** 2019-05-13

**Authors:** Yun Wang, Chenxiao Xu, Ji-Hwan Park, Seonjoo Lee, Yaakov Stern, Shinjae Yoo, Jong Hun Kim, Hyoung Seop Kim, Jiook Cha, The Alzheimer's Disease Neuroimaging Initiative

**Affiliations:** a Department of Psychiatry, Columbia University Medical Center, New York, NY, USA; b Department of Applied Mathematics, Stony Brook University, Stony Brook, NY, USA; c Department of Biostatistics, School of Public Health, Columbia University Medical Center, New York, NY, USA; d Department of Neurology, Columbia University Medical Center, New York, NY, USA; e Computational Science Initiative, Brookhaven National Laboratory, Upton, NY, USA; f Department of Neurology, National Health Insurance Service Ilsan Hospital, Goyang, Republic of Korea; g Department of Physical Medicine and Rehabilitation, National Health Insurance Service Ilsan Hospital, Goyang, Republic of Korea; h Data Science Institute, Columbia University, New York, NY, USA

**Keywords:** Alzheimer's disease, Multimodal MRI, DWI, Machine learning

## Abstract

Accurate, reliable prediction of risk for Alzheimer's disease (AD) is essential for early, disease-modifying therapeutics. Multimodal MRI, such as structural and diffusion MRI, is likely to contain complementary information of neurodegenerative processes in AD. Here we tested the utility of the multimodal MRI (T1-weighted structure and diffusion MRI), combined with high-throughput brain phenotyping—morphometry and structural connectomics—and machine learning, as a diagnostic tool for AD. We used, firstly, a clinical cohort at a dementia clinic (National Health Insurance Service-Ilsan Hospital [NHIS-IH]; *N* = 211; 110 AD, 64 mild cognitive impairment [MCI], and 37 cognitively normal with subjective memory complaints [SMC]) to test the diagnostic models; and, secondly, Alzheimer's Disease Neuroimaging Initiative (ADNI)-2 to test the generalizability. Our machine learning models trained on the morphometric and connectome estimates (number of features = 34,646) showed optimal classification accuracy (AD/SMC: 97% accuracy, MCI/SMC: 83% accuracy; AD/MCI: 97% accuracy) in NHIS-IH cohort, outperforming a benchmark model (FLAIR-based white matter hyperintensity volumes). In ADNI-2 data, the combined connectome and morphometry model showed similar or superior accuracies (AD/HC: 96%; MCI/HC: 70%; AD/MCI: 75% accuracy) compared with the CSF biomarker model (t-tau, p-tau, and Amyloid β, and ratios). In predicting MCI to AD progression in a smaller cohort of ADNI-2 (*n* = 60), the morphometry model showed similar performance with 69% accuracy compared with CSF biomarker model with 70% accuracy. Our comparisons of the classifiers trained on structural MRI, diffusion MRI, FLAIR, and CSF biomarkers showed the promising utility of the white matter structural connectomes in classifying AD and MCI in addition to the widely used structural MRI-based morphometry, when combined with machine learning.

## Highlights

• This study tests the utility of multimodal brain MRI and machine learning in diagnosis and prognosis of AD.
• Models trained on connectomes and morphometry beast classify elders with AD or MCI from cognitively normal elders.
• Models trained on morphometry best predict MCI to AD progression.

## 1 Introduction

There is an urgent, unmet need for clinically useful biomarkers of risk for Alzheimer's disease (AD) based on non-invasive and affordable measures suited for routine examination of individuals with subthreshold symptoms. Studies have focused on brain MRI-derived markers. Cortical thinning and reduced hippocampal volumes based on structural MRI are known for markers for AD, but these structural estimates alone are insufficient for implementation at clinical settings because of insufficient accuracy and generalizability (Teipel et al., 2015).

It is conceptualized that biomarkers of Aβ deposition or neurofibillary tangles become abnormal early, and then markers of neuronal neurodegeneration or dysfunction show abnormality later in AD. These markers of neurodegeneration, rather than those of Aβ or Tau proteinopathy, appear directly related to cognitive symptoms (Jack Jr. et al., 2010). Neurobiology of AD relates to axonal and neuronal degeneration followed by fibrillar lesions triggered by amyloid precursor protein-initiated death-receptor mechanism and activation of tau (Holtzman et al., 2011; Nikolaev et al., 2009). Initial axonal degeneration may lead to grey matter tissue changes and finally to neuronal loss or atrophy resulting in cognitive and functional impairment. Since diffusion MRI uses water molecules as an endogenous tracer to probe tissue microstructure or properties (Beaulieu, 2002), it can detect subtle changes in microstructure tissue properties in AD. Previous studies have shown that decreased white matter integrity is associated with AD (Acosta-Cabronero et al., 2010; Douaud et al., 2011; Zhang et al., 2009).

A potentially powerful application of diffusion MRI to AD research is assessing axonal white matter tracts using tractography. Tractography is a computational estimation of white matter tracts using biophysical modeling of fiber orientations (Johansen-Berg and Behrens, 2006; Seehaus et al., 2013). Recent advances in computational methods have enabled more rigorous estimation of white matter tracts (Azadbakht et al., 2015; Ciccarelli et al., 2008; Shi and Toga, 2017; Sporns, 2011). In AD, human imaging of APP and tau shows widespread topography. Given this, when tractography is applied at the connectome level, the resultant structural connectome estimates could be useful for assessing axonal or white matter abnormalities across the entire connectome. A few studies using tractography at the connectome level have noted abnormal topological organization of structural connectome in AD (Dai and He, 2014; Lo et al., 2010). However, it remains untested whether and to what extent the structural connectomes carry additional information that structural MRI and morphometry do not present.

In this study, we addressed this issue using rigorous, data-driven machine learning techniques in two independent datasets of moderate sample sizes (211 elders for the first dataset [Korean National Health Insurance Service Ilsan Hospital, South Korea] and 179 elders for the second, generalizability dataset [ADNI-2]). In both data, using multi-modal brain MRI (structural and diffusion MRI), we performed high-throughput brain phenotyping, including automated morphometry and white matter structural connectomics (probabilistic tractography) to generate large-scale multi-modal, multi-parametric imaging-derived phenotypes used as features in machine learning. A well-established, rigorous analysis pipeline was applied to diffusion MRI to estimate robust, individualized structure connectomes. We compared data-driven machine learning classifiers trained on the individualized brain connectome and morphometric estimates with benchmark models (white matter hyperintensity) for the first Korean data and CSF biomarkers for the second reproducibility ADNI-2 data) using derived metrics.

## 2 Materials and methods

### 2.1 Participants

For the NHIS-IH Cohort, we used data from 211 seniors who visited the dementia clinic at National Health Insurance Service Ilsan Hospital (NHIS-IH), Goyang, South Korea from 2010 to 2015. This sample is a randomly selected subset of the Ilsan Dementia Cohort, a retrospective clinical cohort. Neurologists made a diagnosis based on possible AD and Peterson's MCI criteria (Petersen, 2004), clinical history, a full battery of neuropsychological evaluations (Seoul neuropsychological screening battery) and MMSE (Mini-Mental State Examination). Those with vascular changes were not excluded from the study as long as they had a diagnosis of AD or MCI. Diagnosis is based on MMSE, CDR, and the neuropsychological evaluations. Distinction between MCI and SMC was based on the full battery of the neuropsychological evaluation (Seoul Neuropsychological Screening Battery-Dementia Version)(Ahn et al., 2010). To meet the diagnosis of MCI, an individual must show a neuropsychological score 1 SD below the normal range at least one of the nine domains of the full battery. Thus, all individuals with SMC show neuropsychological scores within the normal range; they are thus cognitively normal. Those with AD as a primary diagnosis and with small vessel disease were noted as “AD with small vessel disease”. Participants included 110 with the diagnosis of Alzheimer's disease (AD; median age = 82; interquartile intervals (Q3-Q1) = 85–77), 64 with mild cognitive impairment (MCI; median age = 73; Q3-Q1 = 77–66), and 37 subjective memory complaints (SMC; median age = 74; Q3-Q1 = 78–72) (Table 1). To test the generalizability of our approach, we also used structural and diffusion MRI from ADNI-2 (Alzheimer's Disease Neuroimaging Initiative). Demographical information is provided in Table 1. The institutional review board approved this study.

**Table 1:** Participant dmographics.

NHIS-IH Cohort					
	AD (*N* = 110)	MCI (*N* = 62)	SMC (*N* = 36)	Test Statistics	*P* value
Age,Mean (SD)	79.95 (6.61)	71.42 (8.62)	72.25 (6.99)	F = 32.72	*P* < 0.001

Sex					
Female	74	38	32	χ^2^ = 8.56	*P* = 0.014
Male	36	24	4		
Education	6.7 (5.2)	9.8 (4.6)	7.6 (4.9)	F = 6.541	*P* = 0.011
MMSE	18.1 (0.53)	25.1 (0.36)	26.3 (0.37)	F = 151.9	P < 0.001
CDR	1.03 (0.57)	0.54 (0.13)	0.50 (0.11)	F = 79.38	P < 0.001


ADNI-2 Cohort					
	AD (*N* = 48)	MCI (N = 60)	HC (*N* = 71)	Test Statistics	P value
Age,Mean (SD)	74.96 (8.59)	72.57 (6.62)	72.55 (5.66)	F = 3.11	*P* = 0.08

Sex					
Female	20	20	43	χ^2^ = 10.28	*P* = 0.006
Male	28	40	28		
Education	15.31 (2.87)	16.08 (2.68)	16.28 s(2.72)	F = 6.541	*P* = 0.07
CDR	0.82 (0.24)	0.50 (0.00)	0	F = 663.1	P < 0.001

**NHIS-IH,** National Health Insurance Service Ilsan Hospital; **SD**, standard deviation; **MMSE,** Mini Mental State Examination; **CDR**, the clinical Dementia Rating; **ADNI-2,** Alzheimer's disease neuroimaging Initiative.

### 2.2 MRI acquisition

National Health Insurance Service Ilsan Hospital (NHIS-IH): We collected the following multimodal MRI from all participants: T1- MPRAGE: TE, 4.6 ms; matrix, 310 × 480× 480; voxel size, 0.5 × 0.5 × 0.5 mm. T2-FLAIR; matrix = 320 × 240 × 240; voxel size = 0.56 × 1.04 × 1.04. Diffusion MRI: matrix = 112 × 112 × 70; voxel size = 1.9 × 1.9 × 2.0 mm; the series included one image acquired without diffusion weighting and with diffusion weighting along 40 non-collinear directions (b = 600 s/m − 2). ADNI-2: T1-weighted anatomical MRI and diffusion MRI. T1-MPRAGE: TE, min full echo; matrix, 208 × 240× 256; voxel size, 1 × 1 × 1 mm. Diffusion MRI: matrix = 256 × 256 × 46; voxel size = 1.36 × 1.36 × 2.7 mm; the series included 5 image acquired without diffusion weighting and with diffusion weighting along 41 non-collinear directions (b = 1000 s/m − 2).

### 2.3 MRI analysis-structural MRI

The high-throughput computational analysis was conducted. First, we estimated morphometric estimates using the Freesurfer image analysis pipeline (Fischl, 2012) (v6.0) based on T1 and T2-FLAIR images. Morphometric measures (*N* = 948 per subject) include volumes of the hippocampal subdivisions, and thickness, surface area, and volume of cortical/subcortical regions using two different atlases available in Freesurfer (Desikan-Killiany atlas and Destrieux atlas). The technical details of these procedures are described in previous studies (Desikan et al., 2006; Destrieux et al., 2010; Fischl and Dale, 2000; Fischl et al., 1999). In brief, the image processing includes motion correction, removal of non-brain tissue, Talairach transformation, segmentation, intensity normalization, tessellation of the grey matter-white matter boundary, topology correction, and surface deformation. Deformation procedures use both intensity and continuity information to produce representations of cortical thickness. The maps produced are not restricted to the voxel resolution and are thus capable of detecting submillimeter differences between groups.

### 2.4 MRI analysis-diffusion MRI

We estimated the structurals connectome from structural and diffusion MRI. Structural MRI was used to define seed and target nodes of the connectome in each brain. We used the diffusion MRI analysis pipeline, MRtrix 3 (Tournier et al., 2004). The connectome measures (33,698 features per subject) include counts of streamlines, a surrogate measure of structural connectivity (Cha et al., 2015; Cha et al., 2017; Cha et al., 2016), and mean length of streamlines given any two brain regions based on multiple atlases. Diffusion-weighted magnetic resonance imaging (DWI) was preprocessed using the following pipeline in MRtrix 3. DWI was first denoised using a novel algorithm based on random matrix theory that permits data-driven, non-arbitrary threshold for Principal Component Analysis denoising; this method enhances the DWI quality for quantitative and statistical interpretation (Veraart et al., 2016). Denoised images then underwent eddy current and motion correction (Andersson and Sotiropoulos, 2016), brain extraction from three non-diffusion-weighted images (taking their median), and bias field correction using N4 algorithm (N4ITK), an improved N3 method, in Advanced Normalization Tools (Tustison et al., 2010). We then estimated fiber orientation distributions from each preprocessed image using 2nd-order integration over fiber orientation distributions (iFOD2). Based on the FODs, probabilistic tractography was performed using constrained spherical devolution (CSD). We used a target streamline count of 10 million across the whole brain. The tractograms were filtered using spherical-deconvolution informed filtering of tractograms (SIFT) with a target streamline count of 3 million. This method permits mapping to streamline estimation back to individual's DWI and updating a reconstruction to improve model fit. It renders the streamline counts connecting two brain regions proportional to the total cross-sectional area of the white matter fibers connecting those regions, thus enhancing streamline counts as a biologically plausible quantity, representing “structural connectivity”. Finally, from the filtered tractograms, we generated a connectivity matrix in each participant using brain parcellation and segmentation obtained from structural MRI from the same person. In this way, our structural connectome estimates reflect individualized connectomes. We used two different atlases in Freesurfer (Desikan-Killiany atlas (Desikan et al., 2006) and Destrieux atlas (Destrieux et al., 2010). We used streamline counts as the primary connectivity metric in this study as in a recent human infant imaging study (van den Heuvel et al., 2015b), as well mean length as secondary measures. A prior macaque study suggests the validity of streamline counts as an indicator of fiber connection strength, with the number of streamlines significantly correlating with tract-tracing strength in the macaque brain (van den Heuvel et al., 2015a).

### 2.5 Machine learning classification

Given our goal to compare the classifiers trained on the distinct multimodal brain phenotypes,rather than to find a novel machine learning algorithm, we used the following three standard algorithms that have been extensively used in the literature(Abraham et al., 2014; Dimitriadis et al., 2018; Pellegrini et al., 2018): random forest, logistic regression (LR) with L1 and L2 regularization, and support vector machine (SVM) with a linear kernel. Also, given the majority of the prior machine learning classification studies in the AD literature are based on binary classification (Pellegrini et al., 2018), we chose binary classification for better comparison. Machine learning models were trained and cross-validated within each dataset separately. As a common preprocessing step for machine learning estimators, we standardized the imaging derived phenotypes by removing the median and scaling them according to the quantile range (i.e., between the 1st and the 3rd quartile); this method is known to be robust to outliers. Model training and validation were done using nested cross-validation to avoid overfitting due to bias to training data (Cawley and Talbot, 2010; Varoquaux et al., 2017). Nested cross-validation uses a series of train/validation/test set splits: In the inner loop, we trained the model and selected a set of hyperparameters using the training set, then optimized the model with validation set; In the outer loop, we estimated generalization error of the underlying model using test sets. For feature selection, we used the ‘forests of randomized trees’ method, an ensemble method to combine the predictions of base estimators built with a learning algorithm, and then tested whether additional PCA-based dimensionality reduction improved the model or not. For hyper-parameter optimization, we used the grid search method, varying C parameter for SVM and LR classifier, and varying the number of estimators and the minimum samples per leaf for random forest classifier. To measure model performance, we used accuracy, sensitivity, specificity, F1 score, and area under the curve (AUC) in receiver operating characteristic (ROC). In diagnostic classification, we tested six different one-versus-one binary classifications, AD (coded as 1) vs. SMC (coded as 0), AD vs. MCI, MCI vs. SMC, AD only vs. AD with small vessel diseases, AD only vs. MCI, AD only vs. SMC. All the ML analyses were done using scikit-learn, a python library for machine learning (Abraham et al., 2014).

### 2.6 Benchmark models

We used existing biomarkers as benchmark models. First, white matter hyperintensity in the Korean NHIS-IH cohort, and CSF biomarkers in the ADNI-2 cohort. White matter hyperintensity measures were estimated from T2-weighted FLAIR images using Wisconsin White Matter Hyperintensities Segmentation Toolbox (Ithapu et al., 2014). This method uses supervised machine learning methods to segment hyperintense regions and generates normalized effective white matter hyperintensity volume. Second, in ADNI-2 data, we used CSF biomarkers (phosphorylated tau, total tau, AB, ratio of phosphorylated tau/AB, ratio of total tau/AB), whose utility as biomarkers for diagnosis of AD (Olsson et al., 2016), MCI, and progression to AD from MCI (Hansson et al., 2006) has been studied. Furthermore, CSF biomarkers are reported to precede symptom onset of MCI (Moghekar et al., 2013).

## 3 Results

### 3.1 Classification of AD and MCI

In the NHIS-IH Cohort, we tested machine learning classification using the white matter structural connectomes and morphometric estimates in 211 elders at the dementia clinic at the Korean National Health Insurance Service Ilsan Hospital. Age and sex alone showed moderate accuracies: AD/SMC: accuracy = 0.77; MCI/SMC: accuracy = 0.63; AD/MCI: accuracy = 0.72. White matter hyperintensity (WMH) served as a benchmark model, for it has been widely tested in the literature.

**Fig. 1:**
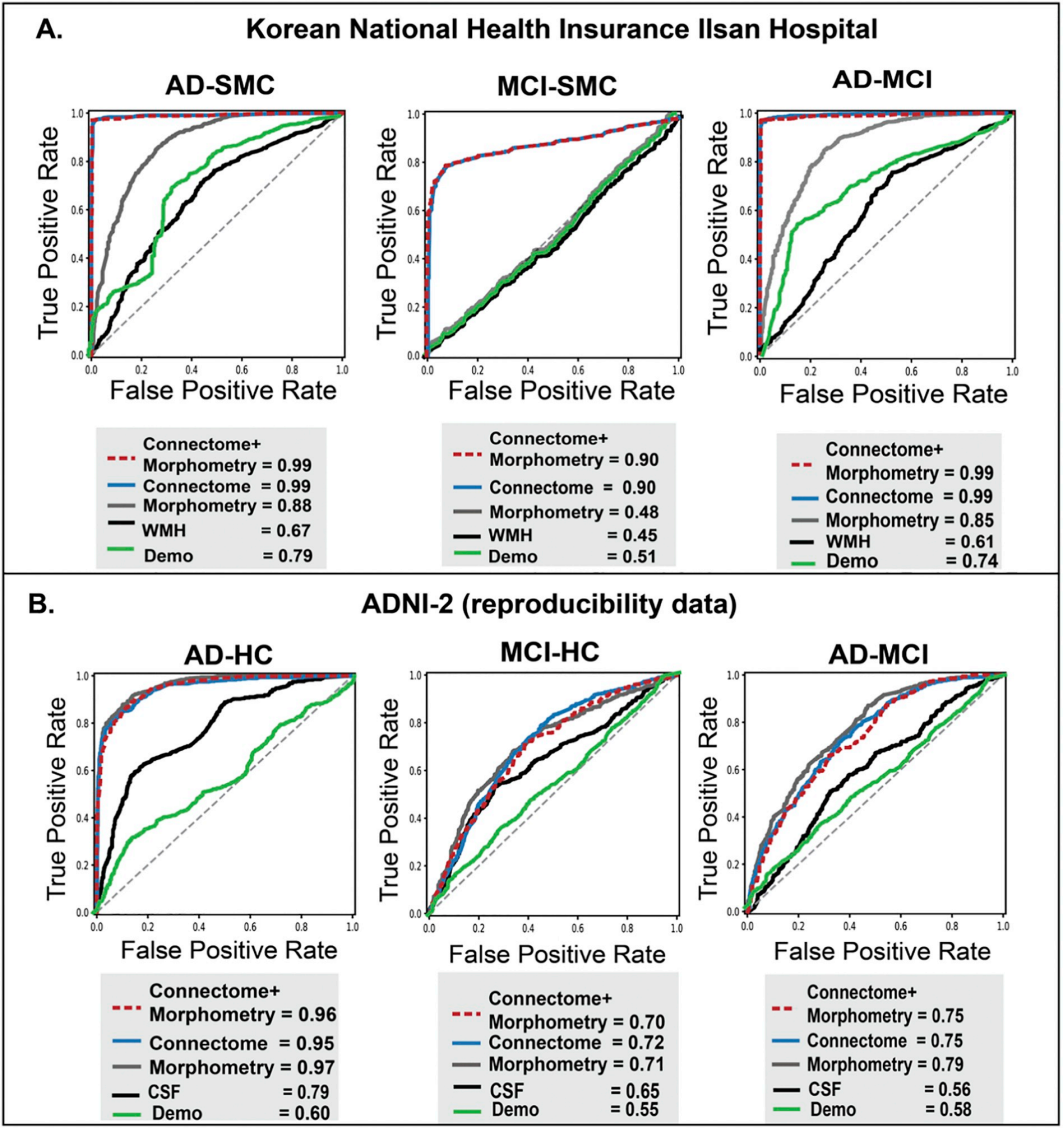
Classification of baseline diagnosis using connectomes and morphometric estimates. Panel (A), classification performances in the NHIS-IH Cohort (Korean National Health Insurance Ilsan Hospital data). It showed higher diagnostic accuracy (area under the curve of the receiver-operator characteristics or AUC ROC) of the machine learning model trained on the connectome and morphometric estimates, compared with the benchmark model trained on white matter hyperintensity. Out of the three machine learning algorithms (random forest, support vector machine, and logistic regression), results from the best models are shown. Panel (B), classification performances in the ADNI-2 Cohort. It showed the reproducible results of diagnostic accuracy of connectomes and morphometry. The combined models show better performance in predicting AD from healthy controls and AD from MCI, and similar in predicting MCI from HC. Results from the best machine learning algorithms are shown. Compared with the NHIS-IH Cohort, the reproducibility data shows less diagnostic accuracy presumably due to multiple sites and stricter inclusion and exclusion criteria in ADNI than in the NHIS-IH study. **WMH**, white matter hyperintensity; **Demo**, demographics including sex, age, and education.

In classification of AD vs. SMC, optimal classification performance was shown in “morphometry+connectome” model (accuracy = 0.97, 95% CI = 0.95–0.98) and “connectome” model (accuracy = 0.97, 95% CI = 0.96–0.98) (Table 2**;** Fig. 1A). These two models outperformed “morphometry” (accuracy = 0.87, 95% CI = 0.85–0.88) and WMH benchmark models (accuracy = 0.73, 95% CI = 0.71–0.75). In classification of MCI vs. SMC, similar classification performance was observed in “morphometry+connectome” (accuracy = 0.82, 95% CI = 0.80–0.85) and “connectome” models (accuracy = 0.83, 95% CI = 0.81–0.85), compared with lower performance of “morphometry” (accuracy = 0.59, 95% CI = 0.57–0.60) and the WMH benchmark models (accuracy = 0.57, 95% CI = 0.54–0.60). In classification of AD vs. MCI, “morphometry+connectome” models showed a best accuracy (accuracy = 0.97, 95% CI = 0.96–0.98), followed by “connectome” model (accuracy = 0.96, 95% CI = 0.95–0.97), “morphometry” model (accuracy = 0.83, 95% CI = 0.80–0.86), and the WMH benchmark models (accuracy = 0.66, 95% CI = 0.64–0.69). Throughout all the classifications, connectomes and morphometry showed greater diagnostic accuracies compared with the WMH benchmark.

**Table 2:** AUC performances of machine learning classifier using structural connectomes, morphometric brain features, and benchmarks.

NHIS-IH Cohort			
	AD vs SMC	MCI vs SMC	AD vs MCI
Morphosmetry + Connectome	0.99(0.99–1.00) ♠	0.90(0.87–0.92) ♠	0.99(0.98–1.00)♠
Connectome only	0.99(0.99–1.00) ♠	0.90(0.88–0.92) ♠	0.99(0.99–1.00) ♠
Morphometry only	0.88(0.86–0.90)	0.48(0.45–0.50)	0.85(0.82–0.88)
Benchmark only (White Matter Hyperintensity)	0.67(0.64–0.70)	0.45(0.42–0.49)	0.61(0.57–0.64)

ADNI-2 Cohort			
	AD vs HC	MCI vs HC	AD vs MCI
Morphometry + Connectome	0.96(0.94–0.97)	0.70(0.67–0.73)	0.75(0.72–0.78)
Connectome only	0.95(0.94–0.96)	0.72(0.69–0.75)♠	0.75(0.73–0.78)
Morphometry only	0.97(0.96–0.98)♠	0.71(0.67–0.74)	0.79(0.76–0.81)♠
Benchmark only (CSF Biomarkers)	0.79(0.77–0.82)	0.65(0.62–0.68)	0.56(0.53–0.59)

**AUC**, area under curve; **NHIS-IH**, National Health Insurance Service Ilsan Hospital; **ADNI-2**, Alzheimer's Disease Neuroimaging Initiative **2; SMC**, subjective memory complaints**; MCI,** mild cognitive impairment; **AD,** Alzheimer's disease; **HC**, healthy control. *All results show mean and standard deviation as **mean** and **95% confidence interval** in this table. ♠ indicates the best models for this classification. For all three classifications, random forest performed as the best classifier, therefore, we only put random forest classifier performance results into this table.

### 3.2 Testing generalizability

We next tested the generalizability of the same multimodal brain imaging-based machine learnings using ADNI-2 data. We included participants in ADNI-2 data whose structural and diffusion MRI (baseline) were collected. To compare the performance of our classifiers, we used the invasive CSF biomarkers (p-tau, t-tau, Aβ42, p-tau/ Aβ42, t-tau/ Aβ42) as a benchmark model. In classifying AD vs. HC, all the MRI-based models showed similarly optimal performance around 0.88 accuracy (Table 2**;** Fig. 1B), outperforming the CSF benchmark model (accuracy = 0.75, 95% CI = 0.73–0.77). In classifying MCI vs. HC, all the MRI-based models showed similar performance with accuracies ranging from 0.64–0.67, outperforming the CSF benchmark (accuracy = 0.62, 95% CI = 0.59–0.65). In classifying AD vs. MCI, all the MRI-based models showed similar performance with accuracy ranging from 0.66–0.71, outperforming the CSF benchmark (accuracy = 0.54, 95% CI = 0.52–0.57) which is barely above chance. These results showed, firstly, morphometry and connectome estimates manifested equally good performance, and consistently exceeded the invasive CSF biomarkers in classifying AD/MCI/HC; secondly, unlike the NHIS-IH results, synergistic effects of combined morphometry and connectomes were not observed using the same machine learning framework.

### 3.3 Testing utility for prognosis

**Fig. 2:**
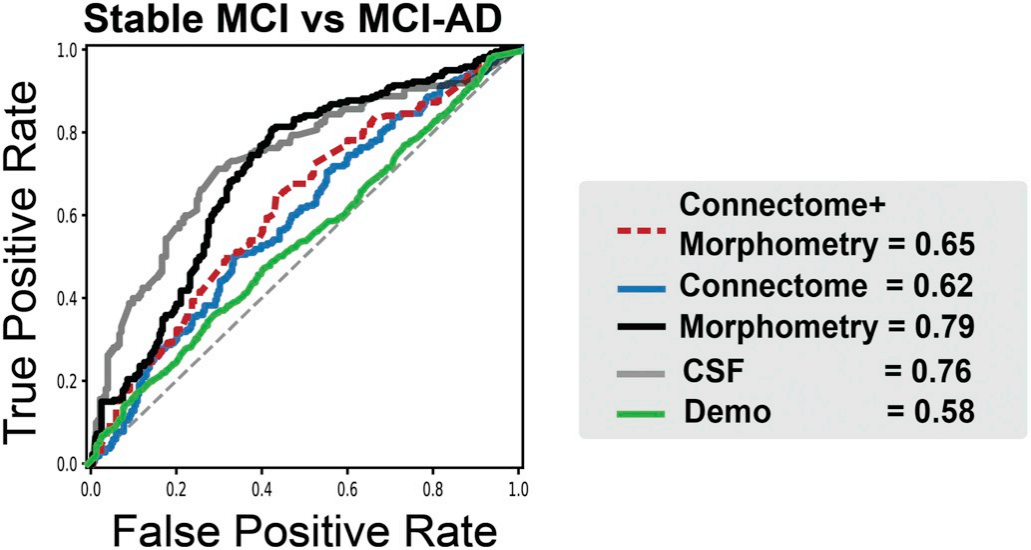
Prediction of progression to AD from MCI using connectomes and morphometric estimates**.** Using ADNI-2 data that has follow-up data after baseline MRI scan, machine learning models were trained on the connectome and morphometry estimates to predict MRI-to-AD progression in 60 elders with MCI (mean follow-up years in stable MCI, 3.76 ± 0.98; range, 2.18–5.32). Morphometry model showed the similar performance to that of CSF benchmark model. Both the combined model and connectome model showed lower but meaningful accuracy.

Of the ADNI-2 data, we further tested the utility of our approach in predicting the disease trajectory. Data from 60 elders were used, whose baseline diagnosis was MCI and who were followed for at least two years. Machine learning models trained on the same five CSF benchmarks were used as a benchmark. In predicting progression from MCI to AD, “morphometry” model showed a highest accuracy (accuracy = 0.69, 95% CI = 0.65–0.73) among MRI-based models, similar to the CSF benchmark model (accuracy = 0.70, 95% CI = 0.66–0.75). (Table 3**,** Fig. 2). “Connectome” model showed a lower, but statistically meaningful accuracy (accuracy = 0.57, 95% CI = 0.53–0.61). Combining the two modalities of morphometry and connectomes (“morphometry+connectome”) did not improve the prognosis accuracy (accuracy = 0.59, 95% CI = 0.56–0.62), compared with “morphometry” model.

**Table 3:** Performance in predicting MCI to AD progression in ADNI-2.

MCI-AD vs. Stable MCI	
Morphometry only (Best: LR + PCA + 20 fold CV)	
Accuracy	0.69 (0.65–0.73)*
Sensitivity	0.79 (0.74–0.83)
Specificity	0.69 (0.64–0.74)
AUC	0.79 (0.74–0.84)
**Connectomes only** (Best: LR + PCA + 20 fold CV)	
Accuracy	0.57 (0.53–0.61)
Sensitivity	0.64 (0.58–0.69)
Specificity	0.53 (0.47–0.59)
AUC	0.62 (0.56–0.68)
**Morphometry + Connectome** (Best: LR + PCA + 10 fold CV)	
Accuracy	0.59 (0.56–0.62)
Sensitivity	0.60 (0.56–0.63)
Specificity	0.68 (0.56–0.79)
AUC	0.65 (0.59–0.71)
**Benchmark: CSF biomarkers** (Best: RF + no PCA + 10 fold CV)	
Accuracy	0.70 (0.66–0.75)
Sensitivity	0.76 (0.72–0.81)
Specificity	0.71 (0.64–0.78)
AUC	0.76 (0.70–0.81)

**ADNI-2,** Alzheimer's Disease Neuroimaging Initiative **2; MCI,** mild cognitive impairment; **AD,** Alzheimer's disease**; LR**, logistic regression; **PCA**, principal component analysis; **CV**, cross-validation. *All results show Mean and standard deviation as **mean** and **95% confidence interval** in this table.

## 4 Discussion

In this study, we used large-scale MRI-derived brain phenotypes (morphometry and white matter structural connectomes) with machine learning techniques to test AD and MCI diagnosis in two independent Alzheimer's disease datasets. We also predicted disease progression to AD from MCI. For high-throughput imaging analysis, we used a well-established automated pipeline for morphometry and a pipeline to estimate rigorously individualized white matter structural connectomes. Firstly, the models trained on morphometry and connectomes showed the best accuracy in classifying AD, MCI, and SMC or HC in the single-site data (ranging from 90% to 99% in AUC ROC; NHIS-IH, South Korea) as well as the multi-site (ranging from 70% to 97% in AUC ROC; ADNI-2, USA) “reproducibility” data. The models outperformed the benchmark models significantly (e.g., white matter hyperintensity or CSF biomarkers) and demographic model (including age, sex, and education). Second, the model trained on connectome or morphometric estimates showed moderate accuracies (ranging from 57% to 79%; AUC) in predicting progression to AD in 60 elders with MCI in ADNI-2 data. These results show the utility of white matter structural connectomes in addition to morphometry in detecting the abnormal brain aging process in AD pathology.

A novel aspect of this study is to assess the utility of the dMRI-based white matter structural connectomes in predictive modeling of AD in a sufficiently large sample (*n* = 211) and to validate it in an independent cohort (*n* = 179). In the NHIS-IH data, the “connectome” model and “connectome and morphometry” model similarly show the optimal classification of AD or MCI, outperforming the benchmark model of white matter hyperintensity. Likewise, in the ADNI-2 generalizability data, both “connectome” and “connectome and morphometry” models show optimal classification accuracy, outperforming the CSF benchmark model. This finding is in line with the literature showing the associations of structural connectomes with potential AD pathology (e.g., topological disturbance based on graph theory) (Pereira et al., 2017) and with healthy aging (Perry et al., 2015). Also, prior studies show the potential utility of connectome estimates in predicting risk for AD, but with a caveat of limited samples sizes (*n* < 30 (Wee et al., 2012; Zhu et al., 2014)). Our study thus further demonstrate the potential practical utility and generalizability of the unbiased brain analytic approach combined with data-driven machine learning, leveraging two independent data with greater sample sizes.

The classification results in the NHIS-IH data may further suggest an important implication. The morphometry model fails to classify MCI from SMC, whereas the connectome or combined model shows optimal classification of 0.90 AUC. The gain of the connectome estimates in classification is more pronounced in MCI/SMC classification than in AD/SMC classification.

This might suggest a greater sensitivity of the white matter connectivity estimates in detecting AD-related neurodegeneration compared with grey matter morphometry. Literature shows the capability of diffusion MRI-derived measures to detect subtle microscopic changes in tissue properties or integrity (Acosta-Cabronero et al., 2010; Beaulieu, 2002; Douaud et al., 2011; Zhang et al., 2009), whereas structural MRI is typically used to estimate macroscopic properties, namely volumes. However, this pattern is not seen in the ADNI-2 multi-site data; this leads to an issue of data harmonization to deal with site effects of MRI-derived estimates.

The connectome or combined model shows ~10% decrease in model performance in the ADNI-2 multi-site data compared with the NHIS-IH single-site data. It is possible that this decrease in performance in the ADNI-2 data is related to the site variability in the dMRI. Indeed, prior studies show persistent inter-site variability in dMRI even when using similar types of scanners, pulse sequences or same field strength (Fox et al., 2012; Mirzaalian et al., 2016). This is a challenging problem because there are hardly any objective ways to assess harmonization of dMRI data (e.g., a dynamic phantom optimized for dMRI). One potential way to mitigate this variability issue across multiple data sources is an analytical solution. A recent elegant study suggests an elegant Bayesian method for post-acquisition harmonization of dMRI (Fortin et al., 2017).

Another potential approach to the MRI harmonization is domain-invariant machine learning. A recent seminal study (Ghafoorian et al., 2017) of white matter hyperintensity segmentation in the brain shows a successful application of “multi-source domain adaption”. That is, a convolutional neural network trained on data from a single domain (i.e., from a single scanner with a single acquisition protocol) was successfully applied (retrained) to the same task with independent MRI from different domains (i.e., different acquisition protocols and image dimension from the same scanner). Given the recent rapid development of the deep learning algorithms, Artificial Intelligence-based domain adaptation may present a promising powerful method of the generalizable and reproducible MRI-based analytics.

In predicting MCI-to-AD progression in the ADNI-2 data, the morphometry model outperforms both connectome and combined models. This may suggest that the grey matter morphometry provides more useful information in predicting the AD trajectory than the connectome estimates. However, given the smaller sample size (*N* = 60) compared with AD/MCI classification (*N* = 119), in this analysis we suspect that machine learning training and feature selection may be suboptimal for the connectome model than for the morphometry model, because of the significantly large number of features in the former (*N* = 33,698) than the latter (*N* = 948). Similarly, while the morphometry model and connectome model respectively showed statistically meaningful (above chance) predictions, when combined, there was little improvement in model performance. This indicates more rigorous methods to combine models trained across multimodal brain imaging-derived phenotypes may be required, such as ensemble methods (Zhang et al., 2011).

Limitations related to the NHIS-IH data include the significantly greater age in the AD group compared with the MCI or SMC groups. It is possible that a greater aging effect embedded on the brain phenotypes may have made the classification of AD easier. However, in ADNI data with the age-matched samples, classification performance (AUC = 0.97) was only slightly less than the NHIS-IH data (AUC = 0.99). This suggests that the patterns extracted from morphometry and white matter connectomes may be specific to AD rather than an age-related bias. Another limitation is the lack of healthy controls in the NHIS-IH cohorts. In this retrospective cohort at the dementia clinic, individuals with Subjective Memory Complaints are cognitively normal. Nevertheless, this group might not be equivalent to healthy controls as in the ADNI data. For example, there might be subtle differences in brain health status between health individuals and cognitively normal individuals with subjective memory complaints. Our study provides no data to address this. However, even if there was a significant difference between cognitively normal SMC in the NHIS-IH data and healthy controls in the ADNI data, it would be a negative bias against the positive classification results. Also, given the fact that in clinical settings, individuals seek for clinical service usually when they suspect symptoms, our results of classifying AD and MCI from individuals with SMC may have a unique clinical utility in addition to the comparisons of AD and MCI with healthy controls in the ADNI data.

In sum, this study lends support for the individualized white matter structural connectomes, estimated from multimodal MRI (structural and diffusion), in combination with machine learning techniques, as a useful method to detect accurately AD-related neurodegeneration across the whole brain in a data-driven manner.

## Appendix A Supplementary data

Supplementary material Image 1
