# Supplementary material for: Diagnosis and prognosis of Alzheimer's disease using brain morphometry and white matter connectomes
Source: Neuroimage Clin. 2019 May 13;23:101859. doi: 10.1016/j.nicl.2019.101859 (PMC6541902; doi:10.1016/j.nicl.2019.101859)
Supplement: Supplementary file 1 — Supplementary material [file mmc1.docx]

**Supplemental Materials**

**Supplementary Table 1. Performances of Machine Learning Classifier using NHIS-IH Structural Connectomes, Morphometric Brain Features, and white matter hyperintensity.**

|  |  | **AD**  **vs**  **SMC** | **MCI**  **vs**  **SMC** | **AD**  **vs**  **MCI** |
| --- | --- | --- | --- | --- |
| ***Morphometry+ Connectome*** | **AUC** | **0.99**(0.99-1.00) ♠ | **0.90**(0.87-0.92) ♠ | **0.99**(0.98-1.00)♠ |
|  | **Accuracy** | **0.97**(0.95-0.98) | **0.82**(0.80-0.85) | **0.97**(0.96-0.98) |
|  | **Sensitivity** | **0.94**(0.92-0.97) | **0.76**(0.72-0.80) | **0.96**(0.94-0.97) |
|  | **Specificity** | **0.98**(0.98-0.99) | **0.87**(0.85-0.90) | **0.98**(0.97-0.99) |
| ***Connectome only*** | **AUC** | **0.99**(0.99-1.00) ♠ | **0.90**(0.88-0.92) ♠ | **0.99**(0.99-1.00) ♠ |
|  | **Accuracy** | **0.97**(0.96-0.98) | **0.83**(0.81-0.85) | **0.96**(0.95-0.97) |
|  | **Sensitivity** | **0.94**(0.93-0.97) | **0.77**(0.74-0.81) | **0.94**(0.91-0.96) |
|  | **Specificity** | **0.98**(0.97-0.99) | **0.88**(0.85-0.91) | **0.98**(0.97-0.99) |
| ***Morphometry***  **only** | **AUC** | **0.88**(0.86-0.90) | **0.48**(0.45-0.50) | **0.85**(0.82-0.88) |
|  | **Accuracy** | **0.87**(0.85-0.88) | **0.59**(0.57-0.60) | **0.83**(0.80-0.86) |
|  | **Sensitivity** | **0.84**(0.79-0.88) | **0.33**♣ | **0.80**(0.75-0.86) |
|  | **Specificity** | **0.88**(0.86-0.89) | **0.63**(0.62-0.64) | **0.85**(0.82-0.86) |
| **Benchmark**  **(White Matter Hyperintensity)** | **AUC** | **0.67**(0.64-0.70) | **0.45**(0.42-0.49) | **0.61**(0.57-0.64) |
|  | **Accuracy** | **0.73**(0.71-0.75) | **0.57**(0.54-0.60) | **0.66**(0.64-0.69) |
|  | **Sensitivity** | **0.38**♣ | **0.26**♣ | **0.44**♣ |
|  | **Specificity** | **0.78**(0.76-0.79) | **0.61**(0.60-0.63) | **0.72**(0.70-0.73) |

**NHIS-IH,** National Health Insurance Service Ilsan Hospital; **SMC**, subjective memory complaints**; MCI,** mild cognitive impairment; **AD,** Alzheimer’s disease.*All results show mean and standard deviation as **mean** and **95% confidence interval** in this table. ♠ indicates the best models for this classification; ♣ indicates the confidence interval is not available. For all three classifications, random forest performed as the best classifier, therefore, we only put random forest classifier performance results into this table.

**Supplementary Table 2. Performances of Machine Learning Classifiers using ADNI-2 Structural Connectomes and Morphometric Brain Features.**

|  |  | **AD**  **vs**  **HC** | **MCI**  **vs**  **HC** | **AD**  **vs**  **MCI** |
| --- | --- | --- | --- | --- |
| ***Morphometry+ Connectome*** | **AUC** | **0.96**(0.94-0.97) | **0.70**(0.67-0.73) | **0.75**(0.72-0.78) |
|  | **Accuracy** | **0.88**(0.86-0.90) | **0.64**(0.61-0.66) | **0.66**(0.64-0.69) |
|  | **Sensitivity** | **0.89**(0.87-0.90) | **0.64**(0.62-0.66) | **0.69**(0.68-0.72) |
|  | **Specificity** | **0.91**(0.88-0.93) | **0.66**(0.62-0.70) | **0.65**♣ |
| ***Connectome***  **only** | **AUC** | **0.95**(0.94-0.96) | **0.72**(0.69-0.75)♠ | **0.75**(0.73-0.78) |
|  | **Accuracy** | **0.89**(0.87-0.90) | **0.64**(0.62-0.67) | **0.67**(0.65-0.70) |
|  | **Sensitivity** | **0.88**(0.87-0.90) | **0.65**(0.63-0.67) | **0.71**(0.68-0.74) |
|  | **Specificity** | **0.92**(0.89-0.94) | **0.66**♣ | **0.67**(0.63-0.71) |
| ***Morphometry***  **only** | **AUC** | **0.97**(0.96-0.98)♠ | **0.71**(0.67-0.74) | **0.79**(0.76-0.81)♠ |
|  | **Accuracy** | **0.89**(0.87-0.91) | **0.67**(0.64-0.69) | **0.71**(0.69-0.73) |
|  | **Sensitivity** | **0.88**(0.86-0.90) | **0.67**(0.65-0.69) | **0.73**(0.70-0.75) |
|  | **Specificity** | **0.92**(0.89-0.95) | **0.69**♣ | **0.70**(0.66-0.73) |
| **Benchmark**  **(CSF Biomarkers)** | **AUC** | **0.79**(0.77-0.82) | **0.65**(0.62-0.68) | **0.56**(0.53-0.59) |
|  | **Accuracy** | **0.75**(0.73-0.77) | **0.62**(0.59-0.65) | **0.54**(0.52-0.57) |
|  | **Sensitivity** | **0.76**(0.74-0.78) | **0.63**(0.60-0.66) | **0.59(0.56-0.61)** |
|  | **Specificity** | **0.76**(0.72-0.80) | **0.61**(0.57-0.65) | **0.49**(0.46-0.52) |

**ADNI-2,** Alzheimer’s Disease Neuroimaging Initiative **2; MCI,** mild cognitive impairment; **AD,** Alzheimer’s disease**; HC**, healthy control. *All results show Mean and standard deviation as **mean** and **95% confidence interval** in this table. ♠ indicates the best models for this classification. For all three classifications, random forest performed as the best classifier, therefore, we only put random forest classifier performance results into this table. ♣ indicates the confidence interval is not available.

Notice: The best classifier for ADonly vs. AD with small vessels is an SVM classifier, and a logistic regression classifier performed best for ADonly vs. MCI, random forest classifier performed best for ADonly vs. SMC.

**Supplementary Table 3 Performances of Machine Learning Classifier using NHIS-IH Structural Connectomes, Morphometric Brain Features, and white matter hyperintensity.**

|  |  | **ADonly**  **vs**  **AD with**  **small vessels** | **ADonly**  **vs**  **MCI** | **ADonly**  **vs**  **SMC** |
| --- | --- | --- | --- | --- |
| ***Morphometry***  ***+ Connectome*** | **AUC** | **0.69**(0.65-0.72) | **0.82**(0.79-0.83) ♠ | **0.80**(0.78-0.82) ♠ |
|  | **Accuracy** | **0.71**(0.69-0.73) | **0.75**(0.72-0.76) | **0.77**(0.76-0.79) |
|  | **Sensitivity** | **0.41**♣ | **0.77**(0.74-0.81) | **0.81**(0.77-0.84) |
|  | **Specificity** | **0.73**(0.72-0.74) | **0.74**(0.72-0.76) | **0.77**(0.76-0.79) |
| ***Connectome*** | **AUC** | **0.64**(0.60-0.68) | **0.79**(0.77-0.81) | **0.78**(0.76-0.80) |
|  | **Accuracy** | **0.70**(0.68-0.72) | **0.74**(0.72-0.76) | **0.76**(0.74-0.78) |
|  | **Sensitivity** | **0.38**♣ | **0.74**(0.71-0.78) | **0.79**(0.75-0.84) |
|  | **Specificity** | **0.73**(0.72-0.74) | **0.75**(0.73-0.76) | **0.76**(0.74-0.77) |
| ***Morphometry*** | **AUC** | **0.69**(0.66-0.72) | **0.78**(0.76-0.81) | **0.80**(0.78-0.82) ♠ |
|  | **Accuracy** | **0.72**(0.70-0.73) | **0.70**(0.67-0.73) | **0.76**(0.74-0.78) |
|  | **Sensitivity** | **0.52**♣ | **0.65**(0.62-0.68) | **0.75**(0.70-0.80) |
|  | **Specificity** | **0.75**(0.73-0.76) | **0.75**(0.72-0.77) | **0.77**(0.75-0.79) |
| **Benchmark**  **(White Matter Hyperintensity)** | **AUC** | **0.96**(0.95-0.97) ♠ | **0.50**♣ | **0.62**(0.60-0.64) |
|  | **Accuracy** | **0.90**(0.87-0.91) | **0.57**(0.56-0.58) | **0.65**(0.64-0.67) |
|  | **Sensitivity** | **0.89**(0.85-0.94) | **0.73**♣ | **0.54**♣ |
|  | **Specificity** | **0.90**(0.88-0.92) | **0.57**(0.56-0.58) | **0.69**(0.68-0.71) |

**NHIS-IH,** National Health Insurance Service Ilsan Hospital; **SMC**, subjective memory complaints**; MCI,** mild cognitive impairment; **AD,** Alzheimer’s disease.*All results show Mean and standard deviation as **mean** and **95% confidence interval** in this table. ♠ indicates the best models for this classification; ♣ indicates the confidence interval is not available. For all three classifications, random forest performed as the best classifier, therefore, we only put random forest classifier performance results into this table.
